# Supplementary material for: Rapid CO2 mineralisation into calcite at the CarbFix storage site quantified using calcium isotopes
Source: Nat Commun. 2019 Apr 30;10:1983. doi: 10.1038/s41467-019-10003-8 (PMC6491611; doi:10.1038/s41467-019-10003-8)
Supplement: Supplementary file 1 — Supplementary Information [file 41467_2019_10003_MOESM1_ESM.pdf]

Supplementary Information

Rapid CO_2 mineralisation into calcite at the CarbFix storage site quantified using calcium isotopes

Philip A.E. Pogge von Strandmann[1*], Kevin W. Burton[2], Sandra O. Snæbjörnsdóttir[3], Bergur Sigfússon[4], Edda S. Aradóttir[4], Ingvi Gunnarsson[4], Helgi A. Alfredsson[3], Kiflom G. Mesfin[3,5], Eric H. Oelkers[1,3,6], Sigurður R. Gislason[3]

[1]LOGIC, Institute of Earth and Planetary Sciences, University College London and Birkbeck, University of London, Gower Street, London, WC1E 6BT, UK.
[2]Department of Earth Sciences, University of Durham, Durham, DH1 3LE, UK.
[3]Institute of Earth Sciences, University of Iceland, Reykjavik, Iceland.
[4]Reykjavik Energy, Reykjavik, Iceland.
[5]HS Orka, Svartsengi, 240 Grindavik, Iceland.
[6]GET, CNRS UMR-5563, Toulouse, France
*Corresponding author email address: p.strandmann@ucl.ac.uk

Supplementary Figures

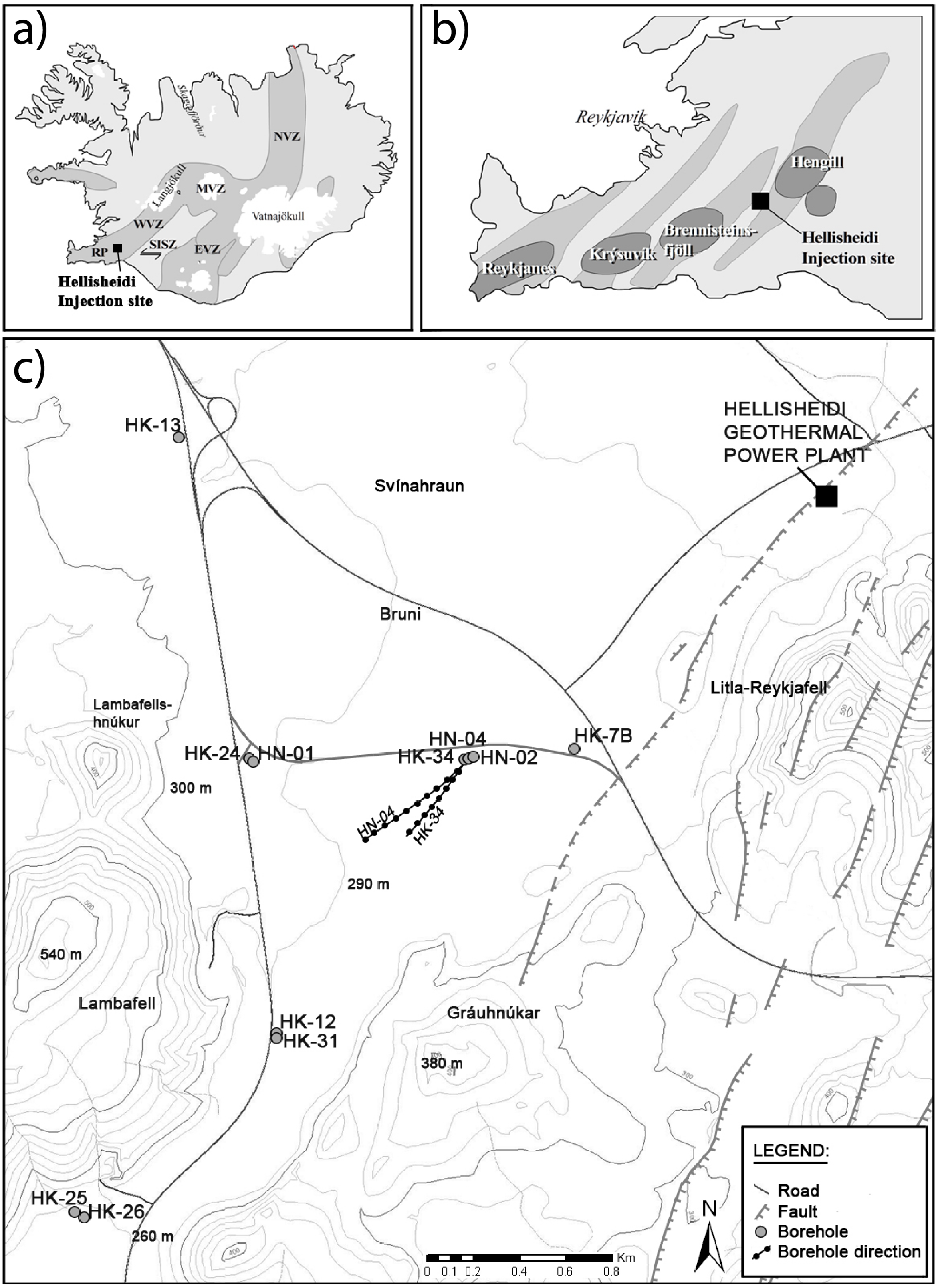

Supplementary Figure 1. Maps showing location of the study area (a) Map showing active volcanic zones in Iceland: RP = Reykjanes Peninsula, WVZ = Western Volcanic Zone, SISZ = South Iceland Seismic Zone, MVZ = Mid-Iceland Volcanic Zone, EVZ = Eastern Volcanic

Zone and NVZ = Northern Volcanic Zone. (b) Map showing location of central volcanoes (dark gray) on Reykjanes Peninsula and associated fissure swarms (gray). (c) Map of the CO2 injection site in, Hellisheidi, SW-Iceland. The CarbFix wells are shown as labeled gray dots. Mapped bedrock faults are located toward east and are part of the Hengill fissure swarm. From Alfredsson et al., 2013[1].

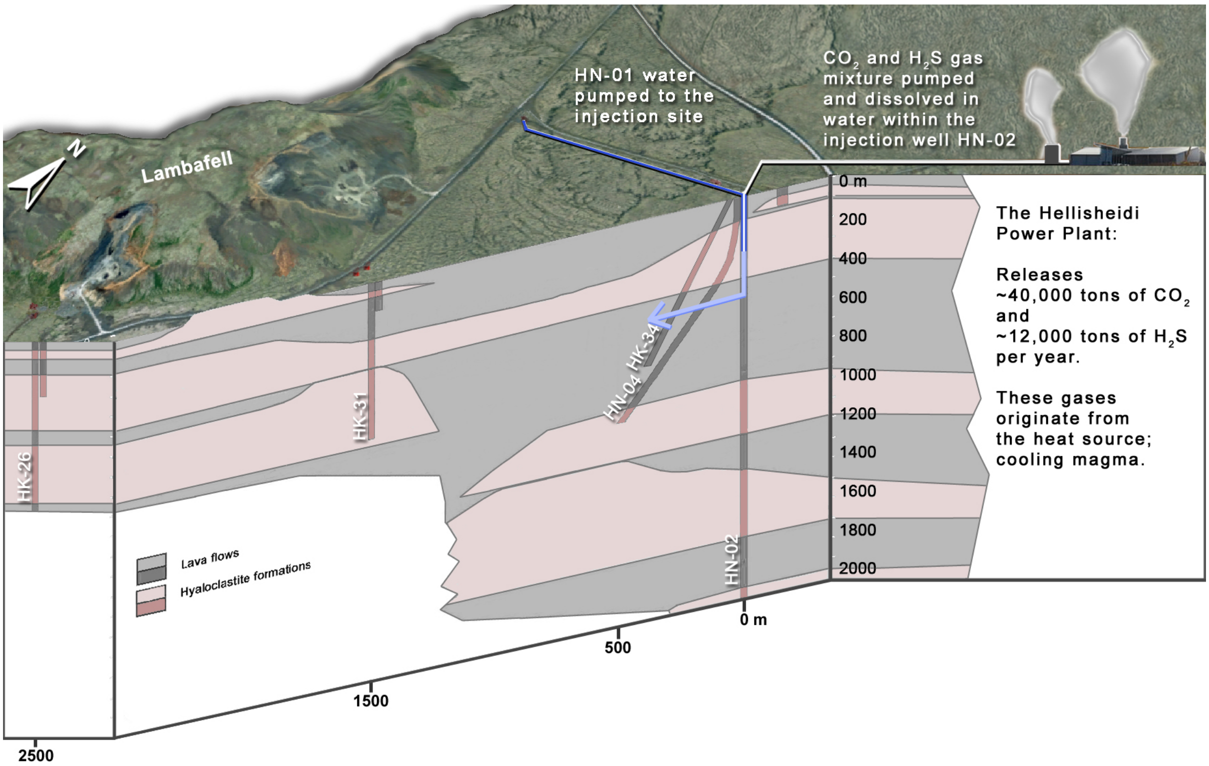

Supplementary Figure 2. Geological cross section of the CarbFix injection site. The CO_2 was co-injected with water from well HN-1 within the injection well HN-2. Modified from Alfredsson et al., 2013.

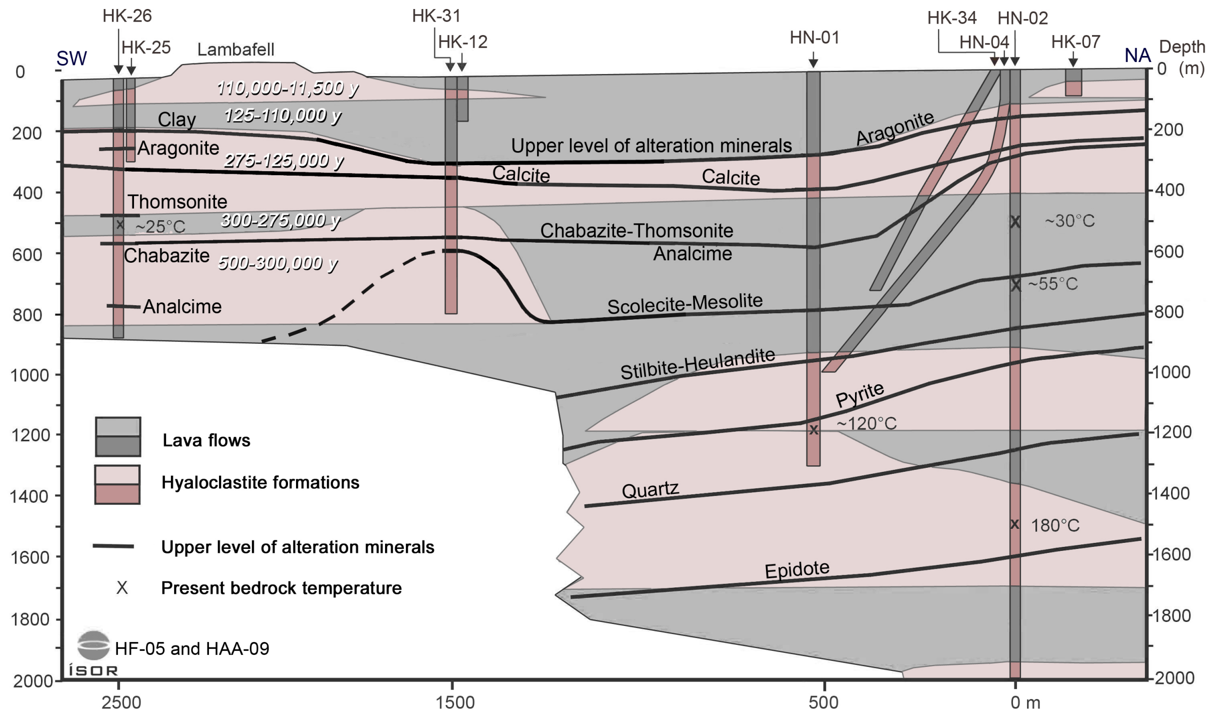

Supplementary Figure 3. Geologic cross section showing the stratigraphy of the CarbFix injection site. The location of the CarbFix wells are shown in the figure; HN-2 is the injection well, HN-1 is the water source well (1 km toward west) and HN-4, HK-34, HK-31, HK-25, HK-7b, HK-12 and HK-25 are monitoring wells. The major stratigraphy consists of hyaloclastite basalt formations (brown) and crystalline basalt formations (gray). Estimated ages of the rocks are provided in the figure. The first appearances of secondary mineral formations in the host rocks are shown as black lines. The bedrock temperature at around 500 m depth is ca. 30°C in HN-2 rising to 180°C at around 1500 m depth. From Alfredsson et al., 2013.

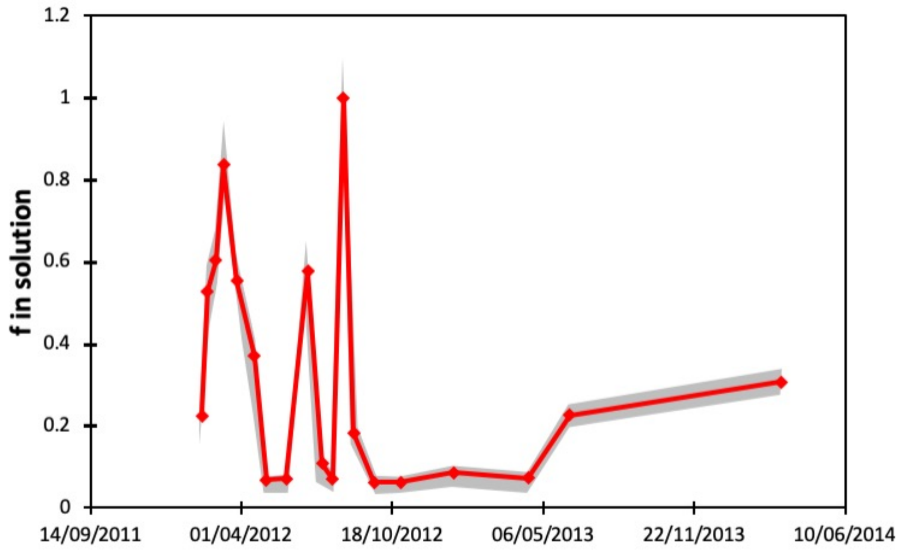

Supplementary Figure 4. Calculated values of *f* with time. The grey shaded areas represent the propagated 2s.e. uncertainty.

1 Alfredsson, H. A. *et al.* The geology and water chemistry of the Hellisheidi, SW-Iceland carbon storage site. *International Journal of Greenhouse Gas Control* **12**, 399-418, doi:10.1016/j.ijggc.2012.11.019 (2013).
